# Is “pre-sepsis” the new sepsis? A narrative review

**DOI:** 10.1371/journal.ppat.1013372

**Published:** 2025-07-31

**Authors:** Rémy Gerard, Antoine Dewitte, Fridolin Gross, Thomas Pradeu, Maël Lemoine, Julien Goret, Maria Mamani-Matsuda

**Affiliations:** 1 Department of Pediatrics, Neonatal Intensive Care Unit, Bordeaux University Hospital, Bordeaux, France; 2 Univ. Bordeaux, ImmunoConcEpT, CNRS UMR 5164, Inserm ERL 1303, Bordeaux, France; 3 Department of Anaesthesia and Intensive Care, Bordeaux University Hospital, Pessac, France; 4 Univ. Bordeaux, Immuno ConcEpT, CNRS UMR 5164, Bordeaux, France; 5 Presidential Fellow, Chapman University, Orange, California, United States of America; 6 Department of Immunology and Immunogenetics, Bordeaux University Hospital, Bordeaux, France; INSERM, FRANCE

## Abstract

Sepsis is a life-threatening condition caused by a dysregulated immune response to infection, leading to organ dysfunction and high mortality. Despite advances in treatment, sepsis remains difficult to manage. Historically, the concept of sepsis evolved from ancient observations of infection-related decay to the germ theory of the 19th century. The latest Sepsis-3 definition describes sepsis as life-threatening organ dysfunction due to a dysregulated host response. However, this clinical characterization may be too late for effective intervention. The concept of endotypes and the ontological data applied to sepsis highlight the substantial heterogeneity in pathophysiological pathways leading to this endpoint. We propose a focus on the “pre-sepsis” phase, where early immune dysregulation arises before significant organ damage. This phase represents the host’s initial response to infection, preceding sepsis and, thus, organ failure. Currently, there is no formal definition of “pre-sepsis”, but this phase could be defined on the basis of early biological pathways in host-pathogen interactions, such as those involving endogenous carbon monoxide. By focusing on “pre-sepsis” and developing tools to detect it, clinicians could intervene earlier and potentially prevent the progression to sepsis. This approach may lead to improved outcomes and more personalized treatments, targeting specific immune pathways tailored to patient profiles. Ultimately, this shift could address existing challenges in sepsis treatment, offering new directions for clinical research and therapeutic development.

## Introduction

Sepsis has undoubtedly existed as long as the time humans and infectious agents have coexisted. However, this complex, life-threatening condition continues to present a formidable challenge for healthcare providers worldwide. Annually, sepsis accounts for approximately 50 million cases and 11 million deaths. It disproportionately affects vulnerable populations in low- and middle-income countries, which account for approximately 85% of sepsis cases and deaths [1]. Sepsis also carries a significant economic burden, with an average hospital-wide cost exceeding 32,000 USD per patient in high-income countries [2]. Despite advances in treatment strategies, including infectious source control, appropriate antimicrobial therapy, and supportive care in intensive care units, sepsis-related mortality remains unacceptably high [1]. Moreover, numerous clinical trials of targeted therapies have yielded disappointing results, highlighting the urgent need to reevaluate the definition of sepsis [3]. The review examines existing definitions of sepsis, critically assesses their strengths and limitations, and proposes a comprehensive framework to guide clinicians and researchers in adopting the most accurate clinically relevant definition of this critical illness. Advancing our understanding of sepsis through an improved operational definition, may open new pathways for early detection and timely intervention, ultimately addressing persistently high mortality rates. To achieve this, we propose a paradigm shift focused on the promising concept of “pre-sepsis”.

## Evolution of the understanding and definition of sepsis

The term “sepsis” originates from the ancient Greek word “σηψις”, meaning the decomposition of organic matter in the presence of bacteria. Its earliest known literary use dates back approximately 2,700 years in a poem by Homer, derived from the verb “sepo”, meaning “I rot” [4–6]. Around 3,000 BC, Ancient Egyptian physicians provided one of the earliest symptomatologic descriptions of sepsis, noting clinical consequences of infection such as fever arising from wounds. Hippocratic literature of the 5th century BC described sepsis as a dangerous biological decay within the body, attributed to humoral dysregulation. Clinically, this decay presented as localized infections “spreading through the vessels”, or and internal lesions leading to purulent collections and auto-intoxication.

The Roman physician Galen (129–199 AD) introduced the concept of “*Pus bonum et laudabile*” (useful and praiseworthy pus), supporting the notion that pus formation is essential for wound healing. Concurrently, Romans believe that sepsis and putrefaction arose from the spontaneous generation of invisible creatures emitting putrid fumes, known as “miasma”. In his work “Canon of Medicine”, Avicenna (980–1037 AD) described the association between blood putrefaction and fever, establishing a foundation for the subsequent understanding of bacteremia and fungemia [7,8]. Despite lacking knowledge of microorganisms, these physicians excelled in describing of the consequences of microbial infections, including sepsis. Notably, they treated their patients with of honey and wine, which have antiseptic properties.

It was not until the 16th century that Galen’s concept and the Roman theory of spontaneous putrefaction were challenged. In his treatise “*De contagione et contagiosis morbis*,” Girolamo Fracastoro (1478–1553) introduced the concept of germ transmission. He proposed that infection results from the transmission of minute, self-replicating bodies with and described three modes of contagion: direct contact, indirectly through fomites, or via the air. This thesis gained support from the first microscopic observations of bacteria in 1647 by Anthony Van Leeuwenhoek [5].

The 19th century marked the golden age of germ theory, with seminal contributions from Semmelweis, Lister, Koch, and Pasteur. Infectious diseases were now attributed to microorganisms present in the environment and capable of transmission. Casimir Davaine (1812–1882), a member of the French Academy of Medicine, conducted experiments that significantly altered the understanding of sepsis. He demonstrated that the injection of putrid blood into rabbit led to its death; blood from that rabbit caused death in another rabbit. The causal agent of this septicemia (now known as sepsis) was designated the “septicemic virus”, and Davaine hypothesized that it could be a vibrion, bacterium, or a toxic substance called sepsine [7,9].

The advent of germ theory validated the pathophysiology of sepsis, establishing it as a recognized disease. This theory also advanced the clinical understanding of sepsis, as evidenced by William Osler’s treatise on sepsis and pyemia in 1892 [10]. Subsequently, Osler emphasized the host response to infection, suggesting that “except on few occasions, the patient appears to die from the body’s response to infection rather than from [the infection itself]” [11]. Hugo Schottmüller reinforced this concept by stating that “sepsis occurs if pathogenic germs invade the bloodstream from an infectious focus and cause symptoms” [12].

The persistent prevalence of sepsis in the latter part of the 20th century, despite the introduction of antibiotics, reinforced the importance of the host response in the pathophysiology of sepsis [13]. Consequently, in 1992, Bone et al. introduced the first widely accepted clinical definition of sepsis as a systemic inflammatory response syndrome (SIRS) resulting from infection (Sepsis-1) [14]. SIRS represents the general and appropriate inflammatory response of the host to various forms of aggression, regardless of its nature. Despite its lack of specificity, the definition of sepsis underwent only minor revisions until 2003 (Sepsis-2) [15], and SIRS was not discarded until 2016.

## Sepsis pathophysiology: The central role of immune system

**Fig 1:**
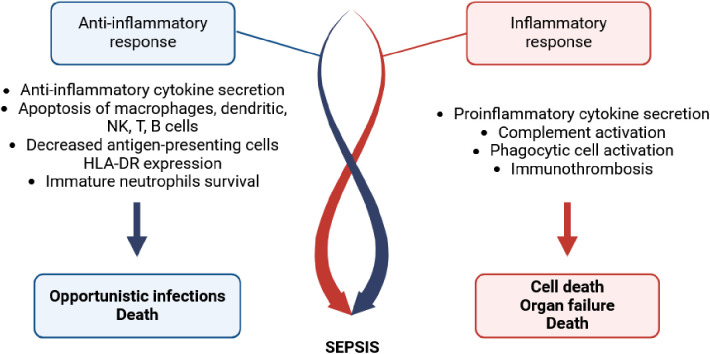
Immunopathogenesis of sepsis. The immunopathogenesis of sepsis involves both pro-inflammatory and anti-inflammatory determinants. These two opposing responses converge, leading to sepsis and potentially death. Created with Biorender.com. https://BioRender.com/rrncrc9.

The pathophysiology of sepsis involves a complex interaction that begins with contact between the infectious agent (typically a bacterium and less commonly viruses or fungi [16]) and the immune system (Fig 1). Recognition of the pathogen’s antigens is primarily mediated by Toll-like receptors, allowing very early activation of the innate immune system [17]. This is followed by a robust inflammatory response, which is mediated and amplified by humoral factors (interleukin-1, interleukin-6, tumor necrosis factor α, complement system, etc.) and phagocytic cells (macrophages, monocytes, and neutrophils) [18,19]. This immune activation leads to abnormalities in vascular tone and permeability, as well as activation of the coagulation system through the induced changes in the endothelial barrier [20]. Neutrophils, monocytes, and complement interact with activated platelets and endothelial cells, resulting in thrombosis in the microcirculation [21]. This phenomenon, termed “immunothrombosis”, contains the infection and suppresses pathogen dissemination [22]. If left unchecked, however, immunothrombosis may become systemic, leading to disseminated intravascular coagulation. The pathophysiology of sepsis involves an early response, immediately after pathogen contact, with intense and excessive activation of the innate immune system in close synergy with the coagulation pathway and endothelium. This pro-inflammatory state can lead to early death from organ failure or later death from persistent inflammation-induced organ damage if balance is not restored [18,23,24].

In addition to this inflammatory response, a compensatory anti-inflammatory response increases susceptibility to opportunistic infections and late death after sepsis [23]. Both the innate and adaptive immune systems contribute to this response. Sepsis has been observed to induce apoptosis in immune cells, including dendritic cells, macrophages, myeloid-derived suppressor cells, and natural killer cells. Conversely, it favors the survival of immature neutrophils with low cytotoxic properties that secrete the anti-inflammatory cytokine interleukin-10. Antigen-presenting cells such as monocytes and dendritic cells, exhibit reduced HLA-DR expression, indicating a diminished capacity to present pathogen-derived antigens to T lymphocytes [24]. In terms of adaptive immunity, sepsis leads to the depletion and apoptosis of CD4 and CD8 T lymphocytes, as well as B lymphocytes. Conversely, regulatory T cells survive and exert immunosuppressive activity. Additionally, the surviving CD4^+^ and CD8^+^ T cells tend to polarize toward an anti-inflammatory type 2 response, rather than an inflammatory type 1 response [24,25].

The precise timing and sequence of these inflammatory and anti-inflammatory events that characterize sepsis remain unclear. Such antagonistic responses are typically depicted as mirror images with temporal symmetry [18,19,23,24]. Hotchkiss et al. also considered their temporal properties, noting that the net immunological response in the early phase favors excessive inflammation, which gradually shifts into “immunoparalysis” [26].

This bimodal immune response is accompanied by non-immunological changes in hormone balance, metabolism, and neurology [27,28]. As part of this response, oxidative stress mediated by reactive oxygen (ROS) and nitrogen (RNS) species sustains excessive inflammatory activation. ROS originate from the activation of innate immune cells (neutrophils, platelets, endothelial cells) and mitochondrial dysfunction induced by pro-inflammatory cytokines, whose secretion is further promoted by ROS [29,30]. When the host’s antioxidant defenses are overwhelmed, ROS trigger cellular and endothelial dysfunction through the modification of proteins, lipids, and nucleic acids [31,32]. Nitric oxide (NO) is an RNS whose production depends on nitric oxide synthase (NOS). Among the three isoforms of this enzyme, inducible NOS (iNOS) is upregulated during sepsis under the influence of pro-inflammatory cytokines such as interleukin-1 and interferon-γ [33]. The NO thus synthesized contributes to the hemodynamic changes observed in sepsis by inducing vasodilation, myocardial dysfunction and decreased sensitivity to vasopressors [33,34]. Endothelium is both a source and a target of NO during sepsis. Endothelial NOS (eNOS) produces large amounts of NO, which contribute to oxidative stress, mitochondrial toxicity, and protein nitrosylation [34].

The resulting endothelial damage further increases vascular permeability and promotes coagulation activation, key processes in the pathophysiology of sepsis that ultimately led to organ failure.

From a pathophysiological perspective, sepsis can thus be defined as a disruption of homeostasis that encompasses both immunological and non-immunological dysregulation. These changes are triggered by contact with an infectious agent and can lead to in organ failure or death. This dysregulated response appears to be dynamic, with components varying over time.

## Is the current Sepsis-3 definition relevant to clinical practice and basic science?

In their 2016 definition of sepsis, Singer et al. introduced a two-dimensional approach [35]. First, they defined sepsis as “life-threatening organ dysfunction resulting from a dysregulated host response to infection”. This conceptual definition is based on the previously discussed pathophysiological components: infection, dysregulated host response, and life-threatening organ dysfunction. Given of the complex and fluctuating nature of the dysregulated host response, they proposed a pragmatic operational definition of sepsis, anchored in the domain of organ dysfunction. This operational definition can be directly applied in clinical settings, using the Sequential Organ Failure Assessment (SOFA) score to assess and quantify organ failure. Thus, we question whether the Sepsis-3 definition is relevant to both clinical practice and basic science.

The most recent clinical guidelines for sepsis management adopt the Sepsis-3 definition, although it is specified that studies are not required to adhere to a specific definition of sepsis to be considered relevant evidence [36]. In clinical practice, a diagnosis of sepsis prompts immediate initiation of treatment, including antibiotic therapy and appropriate hemodynamic support. A precise definition of sepsis is not essential for early and effective management. Recently, pediatric sepsis has been defined based on organ failure, similar to adult sepsis [37]. Thus, it suggests that the definition of sepsis is universal, regardless of patient age or clinical setting.

The revised definition of sepsis emphasizes organ failure, identifying it as the terminal stage of a dysregulated process associated with high mortality. The Sepsis-3 definition replaces that of severe sepsis included Sepsis-2 definition, signifying a shift towards a more severe condition with a worse prognosis [38]. Our current understanding of sepsis aligns with the Hippocrates’ concept of “putrefaction”. By focusing on patients with life-threatening organ dysfunction in clinical and basic science research, we are examining the ultimate consequence of a dysregulated host response rather than the response itself. This gap may explain the prolonged absence of novel therapies for sepsis and the challenges encountered in immunotherapy. Addressing this issue requires that interventions designed for implementation *prior* to sepsis onset. Rather than merely observing the outcomes of an inappropriate host response, it is essential to target the underlying cause.

## Lessons from ontogeny: Sepsis is U-shaped

The age-related incidence of sepsis follows a U-shaped pattern, peaking in the neonatal period and in adults over 60 years of age [1,39,40]. Mortality rates are high at both extremes of life: 10–20% in neonates and 25–35% in older adults. Several points of divergence occur depending on age group (Table 1), starting with the infectious trigger. Early-onset neonatal sepsis (<72 h after birth) is triggered by maternal bacteria transmitted during the peripartum period, such as *Streptococcus agalactiae*, *Escherichia coli*, and *Listeria monocytogenes* [41]*.* After the first 72 h, late-onset neonatal sepsis is primarily caused by bacteria from the newborn’s environment, such as coagulase-negative staphylococci and *E. coli*. In infants, *Neisseria meningitidis* and *Streptococcus pneumoniae* are frequently implicated. A second peak in incidence occurs in adults over 60 years of age; *Streptococcus pyogenes* and *Enterobacteriaceae* are more common in this group. In addition to pathogen type, the initial infection site varies with age. Newborns are more likely to experience primary bacteremia, whereas pneumonia, urinary tract infections, and abdominal infections are predominant in adults.

**Table 1:** Comparative overview of sepsis characteristics by age.

Aspect	Neonatal Sepsis	Pediatric Sepsis	Adult Sepsis	Geriatric Sepsis
Incidence	Up to 350/1000 live births in very premature neonates [40]	About 0,22/1000 population [39]	~2–3/1000 population [1]	Up to 40/1000 population in >85 years [1]
Mortality	10%–20% (up to 50% in preterms) [60]	~10%	~15%–30% (varies by comorbidity) [1]	25%–40%, often with delay diagnosis [1]
Definition	No consensus	Based on Pheonix score	Based on SOFA score	Based on SOFA score
Common pathogens	*S. agalactiae*, *E. coli*, *L. monocytogenes*, CoNS (late onset) [41]	*N. meningitidis*, *S. pneumonia*, *E. coli, S. aureus*, *S. pyogenes* [39]	*E. coli*, *S. aureus*, *S. pneumoniae*, *P. aeruginosa* [16]	Similar to adults, plus MDR organisms, aspiration pathogens [16]
Infection sources	Vertical (early), central lines (late), respiratory [41,60]	Respiratory, CNS, abdominal [39]	Respiratory, urinary, abdominal, bloodstream [8]	Urinary, respiratory, skin, catheter-related [8]
Risk factors	Prematurity, maternal infection, PROM, invasive procedures [50]	Chronic disease, immunodeficiency, devices [39,60]	Comorbidities (DM, COPD, cancer), surgery [28]	Fraitly, immunosenescence polypharmacy [56]
Immune profile	Immature immunity, elevated IL-10, poor antigen presentation [48,50]	Th2-biased, intense IL-10 response [45,47]	Dysregulated inflammation with delayed resolution [24]	Immunosenescence, chronic inflammation [24,52]
Hemodynamics	PPHN, low cardiac output, low NOS [54,55]	Cardiogenic/distributive shock [54]	Distributive ± cardiogenic shock [33]	Blunted CV response, low β-adrenergic reserve [27]
Organ dysfunction patterns	Respiratory distress, PPHN, coagulopathy [60]	CNS involvement, myocardial dysfunction [60]	Multiorgan failure (lung, kidney, liver) [28]	Cognitive dysfunction, AKI, frailty complications [27,56]
Coagulation profil	Low clotting factors, bleeding > thrombosis [60]	Moderate DIC risk [60]	High DIC risk, bleeding and thrombotic [20,21]	High DIC risk, poor reserve [21]
Diagnostic challenges	Nonspecific signs, subtle temperature change [40]	Often atypical, rapid deterioration [39]	Overlaps with chronic disease [28]	Delayed/masked presentation, altered mental status [27,56]
Therapeutic considerations	Empiric antibiotics, immune support [41,60]	Early antibiotics, fluid therapy [36]	Source control, organ support [76]	Avoid deconditioning, stewardship [67,76]

*Abbreviations:* SOFA, Sequential [Sepsis-related] Organ Failure Assessment; CoNS, coagulase-negative staphylococci; MDR, multidrug resistance; CNS, central nervous system; PROM, premature rupture of membrane; DM, diabete mellitus; COPD, chronic obstructive pulmonary disease; IL-10, interleukine-10; PPHN, persistent pulmonary hypertension of the newborn; NOS, NO synthase; CV, cardiovascular; AKI, acute kidney injury; DIC, disseminated intravascular coagulation.

Similar to sepsis in adults [42–44], both pro- and anti-inflammatory cytokines levels increase in pediatric sepsis, contributing to organ failure [45–47]. However, no human studies have directly compared these two age groups. The anti-inflammatory response in the initial phase of pediatric sepsis appears intense, with high plasma levels of interleukin-10 and interleukin-10-receptor antagonist [48]. This response is associated with a worse prognosis [45,47]; such a trend generally is not observed in adults [49]. The immune response is often impaired in early-onset neonatal sepsis [50] or very premature infants [51]. In these situations, a defect in cytokine secretion underscores the importance of immune system maturation *in utero* and during the first days of life.

To differentiate the immune responses of adult and infant populations, numerous studies have utilized animal models. Despite inherent limitations, animal models of sepsis offer insights into the process by enabling manipulation of the infectious trigger. In their study, Gentile et al. examined the immune response to peritoneal infection (cecal slurry) in murine models across distinct age groups: neonates, adults and the elderly. In neonates, the observed influx of immune cells at infection site was lower, as were the secretion of pro-inflammatory cytokines and the production of ROS. The profile substantially differed in the elderly, age group, with enhanced chemotaxis and a hyperinflammatory state. Both extreme age groups showed decreased bacterial clearance and increased mortality [52]. The same study showed an elevated plasma interleukin-10/tumor necrosis factor alpha ratio in neonates, consistent with findings in murine neonatal abdominal sepsis [53].

In addition to the immunological aspects, the pathophysiology of sepsis in children and adults differs in various ways. From a hemodynamic perspective, the pathogenesis of septic shock involves relative or absolute hypovolemia, myocardial dysfunction, and abnormalities in peripheral vascular tone. Although septic shock in adults is typically associated with decreased peripheral vascular resistance and increased cardiac output, in neonates and children it often involves increased peripheral vascular resistance [54,55]. Neonates express lower levels of inducible NOS, which influences peripheral vasodilation in sepsis [56]. Additional developmental data indicate that the myocardium of neonates and children is less capable of compensating for the hemodynamic changes associated with sepsis. Specifically, deficiencies in bathmotropic [57,58], lusitropic [59], and chronotropic functions hinder an adequate response to increased peripheral vascular resistance. The immaturity of the renal, respiratory, and coagulation systems may also worsen neonatal and pediatric sepsis outcomes [60].

This ontogenetic perspective suggests that sepsis can result from different, even opposing processes due to an infectious trigger, that varies according to age.

## The concept of endotype: Not one but many sepsis types

**Fig 2:**
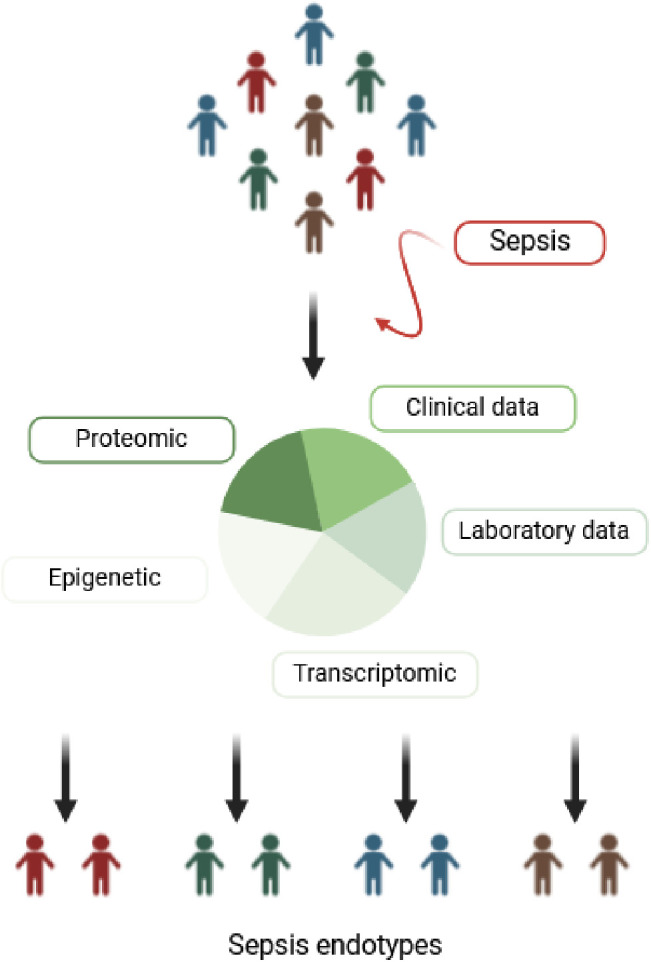
Concept of sepsis endotypes. From a heterogeneous population of sepsis, the concept of endotypes allows the definition of subgroups based on shared pathophysiological characteristics. The goal is to enable earlier diagnosis and propose targeted therapies to improve patient prognosis. Created with Biorender.com. https://BioRender.com/3gj0f10.

The term “endotype” was introduced to address the substantial heterogeneity among sepsis patients (Fig 2) [61], who differ in terms of the pathogens involved, infection sites, and comorbidities. To date, some authors have identified groups of sepsis patients with common gene expression patterns or clinical profiles, enabling prognostic and predictive enrichment [62]. Transcriptomic data have been used to define immunological sepsis phenotypes and to identify patients at the highest risk of mortality (prognostic enrichment) [63–66]. These strategies offer the opportunity to provide personalized therapies tailored to patients’ immune profiles (predictive enrichment). For instance, Fleuriet et al. are conducting a study aimed at identifying sepsis patients who may benefit from corticosteroid therapy based on their immune signature [67].

Several challenges must be addressed before endotypes can be routinely applied in clinical practice. First, the complexity of sepsis pathophysiology is reflected in the multiple existing endotype classifications [62]. Additionally, the concept of endotypes is dynamic, as a single patient’s gene expression can evolve throughout the course of sepsis, as demonstrated by Wong and colleagues [68]. Therefore, simplification efforts are necessary, requiring data sharing and validation in large cohorts. Second, any strategy aimed at improving outcomes in sepsis patients must meet time constraints. Endotype determination appears feasible within hours of patient management, based on a reasonable number of studied genes [69]. Third, the availability and cost of transcriptomic analysis remain barriers to its use in low- and middle-income countries. In these settings, clinical endotypes based on organ dysfunction [70] or temperature curve patterns [71] may provide prognostic and predictive enrichment.

Beyond endotype characterization, some authors emphasize the relevance of studying the gut microbiota in septic patients. Some enterotypes have been associated with an increased risk of sepsis and poorer outcomes [72]. These findings support further investigation of microbiota-targeted preventive and therapeutic strategies, including selective digestive tract decontamination, next-generation probiotics, and fecal microbiota transplantation.

The identification of these sepsis endotypes has the potential to challenge the current singular definition of sepsis by highlighting the existence of multiple forms of this condition. The recognition of these distinct entities can enhance our understanding of sepsis and improve its management. While the current definition relies on the presence of organ failure, which may not capture the full spectrum of sepsis presentations, recognizing different endotypes allows for a more nuanced approach. Elucidation of the molecular and immunological profiles of each endotype can help tailor interventions to address specific dysregulated responses in individual patients. This approach may also enable the identification of biomarkers and the development of targeted therapies to intervene at earlier stages, potentially preventing progression to full-blown sepsis.

## Data-driven discovery of a novel pre-sepsis state

**Fig 3:**
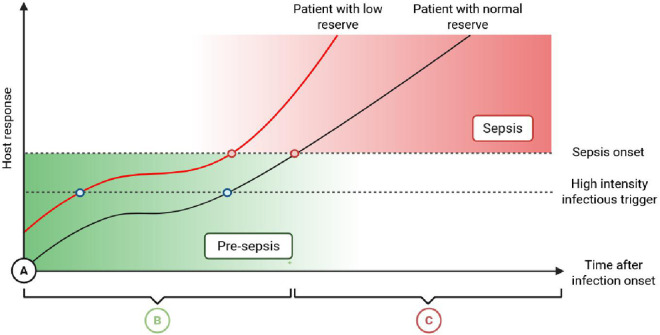
Schematic integration of pre-sepsis in the pathogenesis of sepsis. The pre-sepsis phase **(B)** corresponds to the host response occurring immediately after contact with a microorganism **(A)**. The intensity and duration of this response vary depending host susceptibility and pathogen virulence (blue dots). When the equilibrium point (red dot) is surpassed, sepsis occurs **(C)**, characterized by organ failure. Created with Biorender.com. https://BioRender.com/km9u12y.

Sepsis appears to represent a common outcome of processes originating from various starting points and following distinct pathophysiological pathways. The pathogenesis of sepsis can thus be summarized as shown in Fig 3. After the infectious stimulus, an initial phase, which may be termed as the “pre-sepsis” phase, can be identified. This phase corresponds to the host’s initial response to the infection. As Van der Poll and colleagues suggest, the inflammatory response is at least partially controlled, at this stage, allowing homeostasis to be maintained despite the presence of microorganisms [13]. Accordingly, the onset of sepsis may be prevented at this period, either through the self-limiting nature of the infection or with antibiotic therapy. Importantly, this phase is not simply defined by early immune activation but by the dynamic balance between the intensity of the infectious insult and the host’s ability to maintain physiological stability.

We propose that the “pre-sepsis” reflects a progressive exhaustion of the host’s functional reserves across key physiological systems with individual variability, rather than a uniform threshold applicable to all patients. In this perspective, sepsis occurs when these adaptative capacities are overwhelmed. This concept, drawn from integrative physiology, helps explain interindividual variability in sepsis progression: for instance, older or comorbid patients may reach this tipping point more rapidly than young, healthy individuals. Analogies with effort physiology or myocardial ischemia, where performance or perfusion fails when demand exceeds capacity, illustrate how crossing these individualized limits can lead to decompensation. The nature, duration, and outcome of this pre-sepsis period are thus influenced both by pathogen virulence and host resilience. However, because most studies focus on patients with sepsis defined by overt organ dysfunction, they provide only a retrospective estimate of the processes leading to this state. This represents a key limitation to the concept of endotypes and may partly explain the failure of numerous therapeutic clinical trials in sepsis.

Consequently, research must now prioritize the pre-sepsis phase, aiming to detect, characterize, and define it prospectively in order to better delineate the therapeutic window. This approach requires a shift in perspective, focusing not solely on the endpoint (sepsis), but also on the dynamics occurring immediately after the pathogen-host encounter, before overt organ dysfunction appears. We assume that elucidating these mechanisms will enable the grouping of patients who share common immunological trajectories and vulnerabilities. In this context, the concept of “pre-sepsis” could complement and refine the identification of endotypes. This approach serves three objectives: to improve diagnosis, to stratify prognostic risk, and to support early, personalized therapeutic decisions. We propose developing this innovative framework to expand the current understanding of sepsis.

The term “pre-sepsis” rarely appears in the literature. According to Al-Hassan and Justo, it corresponds to the Sepsis-2 definition and is characterized by the presence of SIRS. Antibiotic therapy is not routinely administered at this stage because there is no consistent progression to sepsis [73]. This view differs from ours: we argue that “pre-sepsis”, by definition will inevitably progress to sepsis and should be addressed prospectively. Furthermore, “pre-sepsis” should be defined based on early immune dysregulation and loss of compensatory capacity, rather than on signs of organ dysfunction, which, by definition, already indicate sepsis.

In their letter to the editor, Bermejo-Martin et al. describe “pre-sepsis” as “a transition from a non-complicated infection to a complicated one”. However, the biological tools they propose are validated primarily in patients who already meet sepsis criteria, thus limiting their utility in the “pre-sepsis” context [74]. It is therefore essential to identify highly specific diagnostic biomarkers, that are detectable soon after the infectious trigger and that reflect early immune dysregulation and loss of compensatory control. Such an approach would make it possible to anticipate clinical deterioration and to intervene before irreversible organ damage occurs. However, this strategy assumes that the patient is already under medical supervision and would be most applicable in hospital or high-risk settings.

To date, the most precise characterization of “pre-sepsis” comes from animal models, where the infectious stimulus is known and controlled. For example, Heithoff et al. conducted a proteomic study in a murine model of sepsis, with measurements between the time of infection and the onset of sepsis. They highlighted coagulation disorders occurring before the development of sepsis [75].

Finally, it is well established that sepsis-related mortality increases significantly with treatment delay [76,77]. Time is therefore a critical dimension of this discussion. The concept of “pre-sepsis” highlights an individualized, time-sensitive window for action, one in which pathophysiological signals may emerge before organ failure, offering a potential turning point for improving outcomes.

## Early biomarkers to identify the pre-sepsis state, example of the CO pathway

Numerous biomarkers have been reported for diagnose sepsis, assess prognosis, and guide early management. These have been extensively reviewed elsewhere [78,79]. A common feature of these biological tools is their strong diagnostic and prognostic performance, as well as their rapid variation following host-pathogen interaction.

Broadly, these techniques can be categorized into those used for bacterial identification, such as molecular biology (DNA amplification), and those aimed at characterizing the patient’s immune system using peripheral blood analysis. The latter includes the evaluation of circulating proteins (e.g., C-reactive protein, procalcitonin, interleukin-6, interleukin-10, tumor necrosis factor-α), the expression of cell surface antigens (e.g., monocyte HLA-DR, neutrophil CD64), blood cell counts, and gene expression profiling through transcriptomics [80]. The rapid turnaround time of these analyses supports their potential use as point-of-care sensors in sepsis management [81].

To be relevant in defining “pre-sepsis”, these biological tools should also be characterized by their ability to reflect dynamic changes over time and identify the point at which the host’s compensatory mechanisms are overwhelmed.

The pathways active during this early phase of the immune response may involve signaling cascades downstream of DAMP and PAMP receptors. In this context, we propose a focus on the CO pathway. Activation of TLR2 and TLR4 during recognition of bacterial antigens has been shown to induce expression of the heme oxygenase-1 (HO-1) gene, responsible for CO synthesis from hemoglobin degradation [82,83]. The secreted CO regulates the inflammatory response by reducing pro-inflammatory cytokines (interleukin-1β, tumor necrosis factor α, interleukin-6) and increasing anti-inflammatory interleukin-10 [84,85]. The CO concentration rises in blood or expired air during sepsis in both murine models and humans, including adults and children [86,87]. Moreover, individuals who survive sepsis exhibit higher CO levels relative to those who do not survive. This finding suggests that CO possesses anti-inflammatory properties that protect severely ill septic patients from further damage and indicates that this marker contributes to the maintenance of immune system equilibrium. Considering these factors, the CO pathway could be a valuable tool for biologically characterizing the “pre-sepsis” state. Furthermore, the potential association between CO levels and patient outcomes makes this biomarker useful for defining endotypes.

## Conclusion

Despite advances in our understanding of the pathophysiology of sepsis, its definition has remained largely unchanged for over two thousand years. The concept of a dysregulated host response to infection was introduced in 2016, but the practical definition of sepsis remains focused on the clinical consequences of this maladaptive process. Treatment of sepsis primarily focuses on managing the underlying infectious trigger and providing symptomatic treatment for organ failure.

To improve the prognosis of sepsis patients, we propose a new paradigm: the “pre-sepsis” state. This theoretical concept marks the initiation of the host response dysregulation in response to infection and requires a concrete biological definition. Establishment of this concept should rely on the most advanced understanding of host-pathogen interactions.

Current research efforts reflect this need for a refined understanding of sepsis pathophysiology. In Europe, the recently established IHU Sepsis Research Center in Paris is dedicated to unravel the mechanisms of sepsis progression and to identify multi-omics signatures of responsiveness to various immune-modulators such as corticosteroids [67,88]. Similarly, large-scale research initiatives in the USA, such as the Sepsis and Critical Illness Research Center (SCIRC) at the University of Florida, are investigating host immune responses, sepsis endotypes and personalized treatment approaches [89]. These projects underscore a growing consensus on the necessity of redefining sepsis through a biological lens, with a particular emphasis on predictive enrichment to optimize treatment response and improve clinical outcomes.

The concept of “pre-sepsis” aligns with that of an endotype because it could facilitate the grouping of patients with a common trajectory into distinct clusters. These hypotheses present promising avenues for therapeutic development—a personalized approach to managing septic patients based on their pathophysiological profiles could be a viable strategy. Predictive enrichment associated with grouping similar patients appears crucial for the execution of clinical trials, mitigating the inherent limitations linked to sepsis heterogeneity.

Future research should focus on multicentric prospective clinical trials to enable the dynamic characterization of individual risks and treatment responses. A major challenge in sepsis research is designing trials that can predict and prevent sepsis, particularly through the early diagnosis of “pre-sepsis”. Developing robust biomarkers and risk stratification models will be essential in identifying patients at risk before clinical deterioration occurs. To establish guidelines for sepsis prevention, future investigations should prioritize integrating real-time patient monitoring, machine learning-based predictive tools, and immunophenotyping into clinical practice.

This novel perspective could serve as a guiding principle for further research, enhancing our understanding of severe infectious states and improving patient outcomes.
